# 212 Multi-trajectory analysis of changes in physical activity, BMI and cardiorespiratory fitness in adolescence

**DOI:** 10.1093/eurpub/ckae114.089

**Published:** 2024-09-26

**Authors:** Iiris Kolunsarka, Michael Beets, Mikko Huhtiniemi, Timo Jaakkola

**Affiliations:** University of Jyväskylä, Jyväskylä, Finland; University of South Carolina, Columbia, United States; University of Jyväskylä, Jyväskylä, Finland; University of Jyväskylä, Jyväskylä, Finland

## Abstract

**Purpose:**

Over the past four decades, the worldwide prevalence of obesity has nearly tripled, with childhood obesity being now more common than ever. Furthermore, children and adolescents with overweight or obesity are at a higher risk of retaining this condition into adulthood (Lobstein et al., 2015). Previous studies have shown that larger increase in body mass index (BMI) during adolescence is associated with less improvements in cardiorespiratory fitness (CRF) and steeper decline in moderate to vigorous physical activity (MVPA) (Kolunsarka et al., 2021). However, the trajectories of these three variables during adolescence are diverse. Therefore, this study aimed to identify and investigate adolescents with similar concurrent changes in MVPA, CRF and BMI throughout adolescence.

**Methods:**

In this four-year longitudinal study (2017-2021), device-measured MVPA, BMI and CRF data were collected annually from 1157 participants (Mage = 11.37 ± 0.37). A multivariate trajectory analysis was used to identify adolescents who share similar BMI, MVPA and CRF trajectories over four years.

**Results:**

Three distinct groups were identified and labelled according to BMI trajectories. The ‘persistently normal’ group (86%) exhibited the highest baseline levels of CRF and MVPA, with significant increases in CRF and a concurrent decrease in MVPA. Conversely, the ‘improving from high’ group (5%) demonstrated a significant increase in CRF and a non-significant (p = 0.07) increase in MVPA. In contrast, the ‘progressing to very high’ group (9%) showed no significant changes in either MVPA or CRF.

**Conclusion:**

These findings underscore the critical importance of early identification and targeted interventions to mitigate the risk of accelerated weight gain, insufficient physical activity and declining fitness during adolescence.

**Funding Source:**

This study was funded by the Strategic Research Council within the Academy of Finland within the project SchoolWell, and the Finnish Ministry of Education and Culture.

**References:**

Lobstein T, Jackson-Leach R, Moodie ML, et al. (2015). Child and adolescent obesity: part of a bigger picture. The Lancet, 385(9986), 2510–2520.

Kolunsarka, I., Gråstén, A., Huhtiniemi, M. & Jaakkola, T. (2021). Development of Children’s Actual and Perceived Motor Competence, Cardiorespiratory Fitness, Physical Activity, and BMI. Med Sci Sports Exerc, 53(12), 2653–2660.

